# Chronic Kidney Disease in Pediatric Recipients of Hematopoietic Stem Cell Transplantation in a 5-Year Observation—A Single Center Experience

**DOI:** 10.1155/ijpe/5335429

**Published:** 2025-10-14

**Authors:** Katarzyna Gąsowska, Katarzyna Zachwieja, Monika Miklaszewska, Aleksandra Krasowska-Kwiecień, Jolanta Goździk, Dorota Drożdż

**Affiliations:** ^1^Department of Pediatric Nephrology and Hypertension, University Children's Hospital of Cracow, Jagiellonian University Medical College, Cracow, Poland; ^2^Doctoral School of Medical and Health Sciences, Jagiellonian University Medical College, Cracow, Poland; ^3^Department of Clinical Immunology and Transplantation, University Children's Hospital of Cracow, Jagiellonian University Medical College, Cracow, Poland

**Keywords:** CKD, hematopoietic stem cell transplantation, HSCT, pediatric population

## Abstract

**Background:**

Chronic kidney disease (CKD) is a common complication of hematopoietic stem cell transplantation (HSCT). However, the data on its risk factors in the pediatric population is conflicting.

**Methods:**

We retrospectively analyzed data from HSCT patients treated between 2005 and 2019, including indications for HSCT, conditioning regimens, and complications observed during a 5-year follow-up period, and calculated estimated glomerular filtration rate (eGFR) values. We used repeated measures ANOVA to model the time course of eGFR as a function of CKD. We built unadjusted and multiple adjusted logistic regression models. We did Kaplan–Meier plots and Cox regression with CKD as a potential risk factor for mortality.

**Results:**

At HSCT, the median age (q5–q95) of the 200 (33.5% female) patients was 8.3 (0.64–17.4) years, median creatinine was 33.8 (17.0–65.5) *μ*mol/L, 49 (24.5%) developed CKD, and 42 (21.0%) died during follow-up. In the unadjusted logistic regression model, CKD was negatively related to baseline eGFR (unadjusted OR per 10 mL/min/1.73 m^2^ greater eGFR: 0.87, 95% CI 0.78–0.95; *p* = 0.004). In the stepwise selection model, greater AKI severity (OR 2.92, 95% CI 1.89–4.15; *p* < 0.0001), presence of hypertension (OR 2.68, 95% CI 1.02–7.05; *p* = 0.05), malnutrition (OR 5.42, 95% CI 1.98–14.82; *p* = 0.001), and iron overload (OR 3.88, 95% CI 1.31–11.51; *p* = 0.01) were related to CKD during follow-up. Female sex was protective (OR 0.19, 95% CI 0.06–0.61; *p* = 0.005). CKD status during follow-up was not related to the risk of mortality.

**Conclusion:**

Our results underline the need for better nutrition, prevention of iron overload, and adequate blood pressure control to prevent CKD in pediatric patients after HSCT.

## 1. Introduction

About 70 years ago, hematopoietic cell infusions were introduced in research and clinical practice, first in animals and then in human subjects [1–3]. Currently, both allogenic and autologous hematopoietic stem cell transplantations (HSCTs), commonly referred to as bone marrow transplants, have become established therapies in numerous medical conditions. These range from malignancies to bone marrow insufficiency–related disorders, through inborn errors of metabolism, among others. The advent of such techniques as gene therapy of hematopoietic cells is broadening the spectrum of further possibilities. Importantly, no absolute contraindications to the procedure can be found if only a patient could be supposed to benefit from this treatment. Since the first successful allogeneic bone marrow transplant was reported in the United States in 1968 for a pediatric patient with severe combined immunodeficiency syndrome [4], a total of 1.5 million HSCTs worldwide were performed by 2019 [5]. Transplants in pediatric patients constitute approximately 30% of all recipients [6].

Even though the last two decades witnessed a marked decrease in transplant-related morbidity and mortality, HSCT still carries a significant risk of complications. Chronic kidney disease (CKD) is often reported among these [7]. However, the data regarding the incidence of CKD post-HSCT, the factors associated with increased risk of renal involvement, and the potential influence of post-HSCT CKD on survival in the pediatric population are conflicting.

Based on data from a large population of pediatric HSCT recipients followed in a single high-volume center, we aimed to analyze the incidence of CKD, investigate factors associated with the incidence of CKD, check the evolution of estimated glomerular filtration rate, and finally check whether post-HSCT CKD is associated with poorer survival over 5 years of follow-up.

## 2. Materials and Methods

We retrospectively gathered data of all pediatric patients (< 18 years of age at the time of the hematopoietic cells transfusion, 200) who underwent allogenic HSCT between January 2005 and August 2019 at the Department of Clinical Immunology and Transplantation, Polish-American Pediatric Hospital, Jagiellonian University Medical College, Kraków, Poland.

Based on the electronic records, we obtained their baseline medical history, including the indications for the HSCT, baseline renal parameters (serum creatinine, cystatin C, and urea concentrations), complete blood count, serum albumin, and total protein concentrations. We obtained the initial 5 years post-HSCT annual follow-up data concerning survival, renal function, development of AKI, CKD, and acute and chronic graft-versus-host disease (GVHD) and information concerning the complications such as infections (sepsis, *Clostridium difficile*, viral including cytomegalovirus [CMV], and fungal), development of DM, liver dysfunction, including venoocclusive disease/sinusoidal obstruction syndrome (VOD/SOS), and pancreatitis.

We used the values outside the following ones to diagnose thyroid disease: thyroid-stimulating hormone (TSH) normal limits: 0.9–7.7 mIU/L in children < 1 year old, 0.4–6.0 mIU/L in patients at prepubertal age, 0.5–4.8 mIU/L in teenagers, free triiodothyronine (FT3) 3.5–6.5 pmol/L, and free thyroxine (FT4) 12.0–22.0 pmol/L.

All above were diagnosed according to local guidelines criteria specific for each disease [8, 9, 10, 11]. Infections were confirmed after positive microbiological tests. Adrenal insufficiency was extracted from patient records where it was defined based on clinical status, electrolyte imbalances, and low cortisol for which the consulting endocrinologist prescribed adrenal hormonal supplementation. Clinical diagnosis of malnutrition was extracted from patient records, where it was based on low height or weight for age (< 3rd centile) or a low BMI (< 10th centile), as applicable. Iron overload was diagnosed based on the elevation of iron, ferritin, and total iron-binding capacity (TIBC) above the age-specific values (serum iron concentration: < 2 years old > 25 *μ*mol/L, 2–18 years > 26 *μ*mol/L, and > 18 years females > 30 mol/L and males > 32 *μ*mol/L; serum ferritin: 1–2 months old > 600 *μ*g/L, 3–5 months > 200 *μ*g/L, 6 months–15 years > 140 *μ*g/L, and > 15 years females > 103 *μ*g/L and males > 306 *μ*g/L; and TIBC: < 2 months > 42 *μ*mol/L, 2–4 months > 69 *μ*mol/L, 4 months–18 years > 70 *μ*mol/L, and > 18 years > 71.2 *μ*mol/L) [12, 13].

The eGFRs (pre-HSCT, at the time of HSCT, then at 6, 12, 24, 36, 48, and 60 months post-HSCT) have been calculated based on the Revised Bedside Schwartz formula from eGFR calculator provided by National Kidney Foundation [14], which enables estimation of GFR across a wide age and body-build ranges. We based the diagnosis of CKD on eGFR or proteinuria or urinary abnormalities or abnormalities detected in imaging studies. Regarding the eGFR threshold, we used the criterion of CKD provided by KDIGO 2024 Clinical Practice Guideline for the Evaluation and Management of CKD [15]. According to that document, children (2 years of age or more) as well as young adults should have eGFR ≥ 90 mL/min/1.73 m^2^.

The study has been approved by the Jagiellonian University Committee for Research Ethics (118.0043.1.16.2025).

Statistical analysis was performed with SAS 9.4 (SAS Institute Inc., Cary, NC, United States).

Normally distributed continuous variables were presented as means (SD), and those significantly departing from the assumption of normal distribution as medians with 5th and 95th percentiles and were compared using Student's *t*-test or Wilcoxon's test, respectively. Differences of eGFR between baseline and follow-up were presented as means (95% CI). Categorical variables were presented as percentages and compared with chi-square test. In the case of ordinal variables with more than two classification levels, we additionally used the Cochran–Armitage test for linear trend. To identify variables potentially associated with the subsequent development of CKD, we searched for the differences between patients who developed or did not develop CKD. We adopted a *p* value of < 0.10 as a threshold for the inclusion of a variable in further analyses. In the next step, we fitted a logistic regression model, with CKD as a response variable and with a stepwise selection of baseline explanatory variables from among those that were indicated in the previous step. We forced age and gender into the model. Using repeated measures ANOVA approach, we checked the between- and within-individual differences in the eGFR as a function of time and CKD status. We plotted the Kaplan–Meier curves of survival given the CKD status and calculated the log-rank *p* value. We used the Cox regression to model time to development of CKD as a function of identified confounders and to model time to death as a function of CKD with adjustment for potentially confounding variables. All *p* values are two-tailed, and we adopted the 5% threshold for significance.

## 3. Results

### 3.1. Characteristics of the Study Group

**Figure 1:**
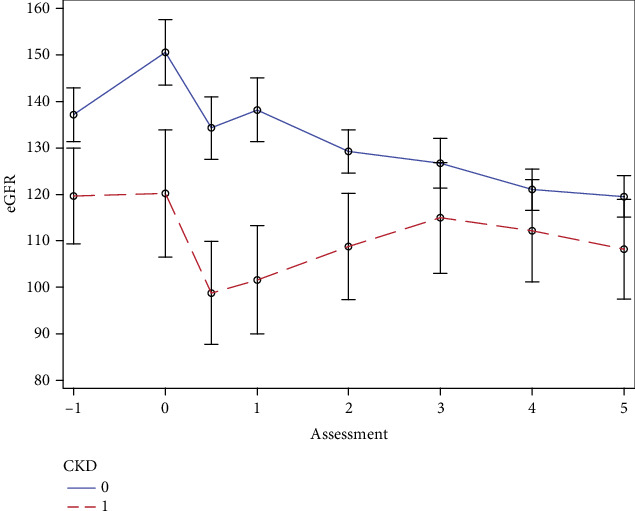
The mean (95% CI) eGFR in patients with and without CKD during follow-up. Timeline indicates pre-HSCT (−1), HSCT (0), 1/2, 1, 2, 3, 4, and 5 years of post-HSCT follow-up.

We included data of all available patients who met the criteria to enter the analysis, which rendered a sample of 200 patients. The median (p5–p95) age of the 133 (66.5%) boys and 67 (33.5%) girls was 8.3 (0.64–17.4) years. The median (p5–p95) body weight of the patients was 27.5 (6.5–17.4) kg. Baseline renal parameters were as follows: median (p5–p95) creatinine 33.8 (17.0–65.5) *μ*mol/l, mean (SD) cystatin C 0.77 (0.40) mg/L, and mean (SD) urea 3.6 (1.6) mmol/L. Apart from the concentration of urea, these parameters did not differ according to the follow-up CKD status (Table 1). Both pretransplantation, transplantation, and annual follow-up eGFRs up to the 3rd year of follow-up were significantly lower in patients who, during follow-up, developed CKD (all *p* < 0.04) (Figure 1). At the pretransplantation stage, 20 (10%) patients had eGFR < 90 mL/min/1.73 m^2^ and four (2%) patients had the pretransplantation eGFR < 60 mL/min/1.73 m^2^. The pretransplantation mean (SD) albumin 36.2 (6.7) g/L and total protein 60.9 (9.5) g/L did not differ according to follow-up CKD status. The characteristics of the study group according to follow-up CKD status are contained in Table 1.

### 3.2. Characteristics Related to Bone Marrow Transplantation

The transplantation-related characteristics did not differ between the patients who during follow-up did or did not develop CKD (Table 2). There was no statistical difference in the frequency of related and unrelated donors among the CKD patients (*p* = 0.28). The bone marrow predominated as a source of cells. The diagnoses that were the indication for the HSCT included leukemia in 48.0%, bone marrow insufficiency in 20.0%, lymphoma in 4.5%, and other causes in 5.0% of the patients. Neither the use of medications nor the total body irradiation for host bone marrow ablation differed between CKD and non-CKD patients (Table 2). The immunosuppressive therapy was also similar in the two groups (Table 2).

### 3.3. Complications During Follow-Up

During follow-up, 157 (78.5%) patients developed acute GVHD. The Glucksberg score did not differ according to the follow-up CKD status (Cochrane–Armitage *p* for trend = 0.14). Chronic GVHD occurred in 93 (46.5%) patients, and the incidence did not differ between the two groups. Thirty-five patients in the follow-up CKD group (71.4%) and 84 patients in the non-CKD group (55.6%; *p* = 0.05) developed a viral infection (Table 1). A significantly greater percentage of patients who developed CKD during follow-up had the CMV infection (42.9 vs. 24.5%, *p* = 0.01). Twenty-two patients took ganciclovir, of whom 14 were in the follow-up CKD group. A similar proportion of patients in the CKD and non-CKD groups developed sepsis (27.5%), required hospitalization in the ICU (23.5%), had heart failure (3%), pancreatitis (acute or chronic, 12.0%), had venoocclusive liver disease (5.5%), or developed elevation of liver function tests (47.5%). More patients with CKD during follow-up had diabetes mellitus, iron overload, adrenal insufficiency, hypothyroidism, malnutrition, and CMV infection (Table 3).

### 3.4. Outcome: CKD, Renal Function, and Mortality During 5-Year Follow-Up

Forty-nine (24.5%) patients, including 41 during the first, six during the second, and two during the third year post-HSCT, developed CKD. Based on the eGFR at respective time points, at the end of the first year of follow-up, 19 patients had Stage 1, 13 had Stage 2, three had Stage 3a, and two had Stage 3b of CKD, and four patients had died. At the third year of follow-up, after which no new cases of CKD accrued, 30 patients had Stage 1, nine had Stage 2, and one had Stage 3b of CKD, and cumulatively nine had died. Overall, at the fifth year of follow-up, 29 patients had Stage 1, seven had Stage 2, two had Stage 3a, and one had Stage 3b of CKD. After 5 years of follow-up, in 39 surviving patients with CKD, eGFR decreased on average by 13.5 mL/min/1.73 m^2^ (95% CI −23.1 to −3.6 mL/min/1.73 m^2^, *p* = 0.009) (Figure 1). The greatest fall in eGFR was observed over the first year of follow-up. Of 49 CKD patients, 45 were alive after 1 year, and they experienced a fall of eGFR of 20.4 mL/min/1.73 m^2^ (95% CI −34.0 to −6.9 mL/min/1.73 m^2^, *p* = 0.004). Ten patients with CKD died in the course of the 5-year follow-up. The median (p5–p95) time from HSCT to the development of CKD was 130 (92–541) days. During follow-up, significantly more patients with subsequent CKD had a diagnosis of AKI, and there was a significant trend for worse KDIGO classification in that group (Cochran–Armitage *p* for trend < 0.0001) (Table 3). No patients in our group required chronic renal replacement therapy.

In the first hierarchically built logistic regression model, unadjusted for potential confounders, CKD was negatively related to baseline eGFR (OR per 10 mL/min/1.73 m^2^ greater eGFR: 0.87, 95% CI 0.78–0.95; *p* = 0.004); this held in the second model after adjustment for sex and age.

In the logistic regression model where age, gender, and pretransplantation eGFR were offered together with other variables that in univariate analyses differed significantly according to CKD status, baseline eGFR was not related (*p* = 0.42) to the development of CKD. Finally, we fitted two stepwise regression models where the same set of variables was used.

In the first, free selection model, greater AKI severity by one grade according to KDIGO (OR 2.92, 95% CI 1.89–4.15; *p* < 0.0001), presence of hypertension (OR 2.68, 95% CI 1.02–7.05; *p* = 0.05), malnutrition (OR 5.42, 95% CI 1.98–14.82; *p* = 0.001), and iron overload (OR 3.88, 95% CI 1.31–11.51; *p* = 0.01) were related to a greater probability of diagnosing CKD during follow-up. In the same model, female sex was associated with less risk of CKD (OR 0.19, 95% CI 0.06–0.61; *p* = 0.005).

In additional model, where gender, age, and baseline eGFR were forced and the selection was carried out from among the remaining pool of variables, hypertension was not associated with the probability of follow-up CKD diagnosis and thus did not remain in the finally optimized set of variables. Additionally, also in this model, lower baseline eGFR was not related (*p* = 0.39) to greater probability of CKD during follow-up.

In the repeated measures ANOVA model, CKD (*p* = 0.02), AKI (*p* = 0.002), and iron overload (*p* = 0.04) were related to the between-individual differences in the eGFR over the follow-up. Of the variables offered to enter the model, only CKD accounted for the gradual decline of GFR (*p* for CKD∗time interaction = 0.04, Figure 1).

Similarly constructed Cox regression model yielded confirmatory results (Table 4), with diabetes mellitus emerging as an additional significant predictor of the development of CKD (Table 4).

**Figure 2:**
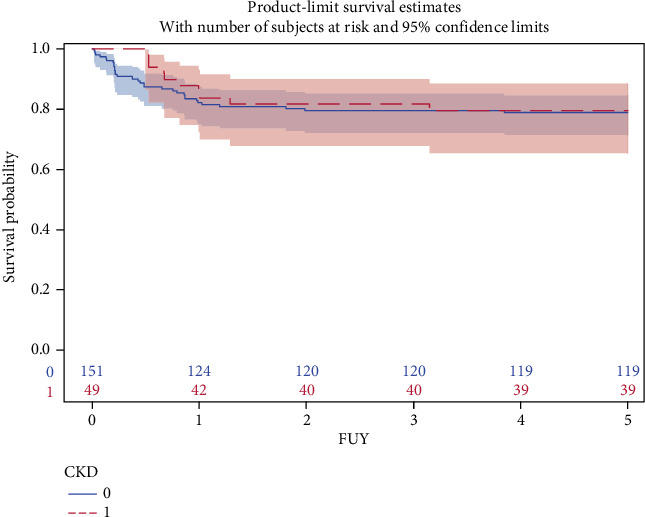
Kaplan–Meier curves, with 95% CI, depicting all-cause mortality according to CKD status during 5-year follow-up after HSCT. Log-rank *p* = 0.79. Caption: Numbers indicate persons at risk at given time. FUY, follow-up (years).

During the 5-year follow-up, 42 patients died. CKD status during follow-up was not related to the risk of mortality (unadjusted log-rank test, *p* = 0.80; Figure 2).

## 4. Discussion

We demonstrated that during the 5-year follow-up after HSCT, approximately a quarter of patients develop CKD and approximately one in five dies; this, however, was not directly influenced by CKD. The factors strongly associated with follow-up CKD include AKI (both overall and more advanced according to KDIGO classification), malnutrition, hypertension, diabetes mellitus, and iron overload (defined by laboratory criteria). Female gender was protective. In the multivariate stepwise logistic regression, none of the factors associated with the conditioning regimen, infection, or treatment in the ICU setting were related to the development of CKD; however, in a univariate analysis, a CMV infection had been more frequent among the patients in whom CKD developed. During the 5-year follow-up, CKD was the factor strongly differentiating the time course of the change in eGFR.

Several studies in adults showed that post-HSCT CKD develops in 23% to even 89% [16, 17]. The factors associated with the development of CKD included TBI, myeloablative conditioning regimen, and GVHD.

Compared to our analysis, studies performed in the population of pediatric patients were smaller [18–26], had shorter follow-up time [18, 19, 23, 26, 27], and included only specific patients [20], often focusing on patients with a narrower range of initial diagnoses [19, 22, 23].

The previously reported prevalence of CKD in post-HSCT children also varied between studies, with the estimates ranging from less than 10% [28, 29] to over 60% [27] and up to 77% [26]. A systematic review performed by Ellis et al. estimated the prevalence at approximately the same level as we found it in our group [6]. The differences may be the function of both different definitions, different follow-up time, and differences in factors influencing the incidence of CKD post-HSCT [30]. Importantly, the reports in the literature vary considerably with respect to the calculation of eGFR. In our analyses, we used the bedside Schwartz formula, which is probably the most frequently used but is not free from the possibility of inaccuracies.

During the 5-year follow-up of our cohort, most of the CKD developed early, during the first year post-HSCT. We based our diagnosis of CKD on decreased eGFR, proteinuria, or abnormalities in kidney imaging studies. This may in part account for our observation that after the adjustment for other confounders, baseline eGFR did not predict the development of CKD. Overall, the eGFR of our patients mostly exceeded 90 mL/min/1.73 m^2^.

In our study, we found that AKI, malnutrition, hypothyroidism, adrenal insufficiency, and hypertension were associated with a greater risk for CKD and that female gender was protective. Hypertension [18, 22, 23, 31, 32, 33] has been linked to a higher risk of CKD post-HSCT, and AKI [25–27, 31, 34, 35] was found to be invariably connected with the development of subsequent CKD. The finding of the association between diabetes and CKD is not surprising. Our study was not designed to address the reasons for diabetes mellitus. However, the use of steroids in HSCT patients must have played a significant role. Diabetes has been widely recognized as an important risk factor for the development of CKD both in adults and pediatric patients [11]. Our finding of gender influence needs separate confirmation and a pathophysiologic explanation. Malnutrition, a multifactorial condition in susceptible individuals, may lead to organ failure, including renal dysfunction [34].

Preserved adrenal and thyroid hormonal function are crucial to the maintenance of metabolism in health and disease [34]. Dysfunction of oxygen metabolism and the drop-out of the multifaceted adrenal cortical function, especially under the stress exerted by a disease requiring HSCT and by the procedure itself, are among factors likely to have an influence on renal function [36, 37].

Our finding that iron overload was associated with the development of CKD may be reflecting the mostly hematologic indications for HSCT and the need for repeated transfusions of packed red blood cells [13, 38] or increased iron release from damaged body tissues and absorption through inflamed mucosa of the gastrointestinal tract [39, 40]. To the best of our knowledge, no other study to date examined the connection between iron overload and the subsequent development of CKD in pediatric recipients of HSCT, and the mechanism has not been explained yet. Studies in patients with CKD developing iron overload postulated that inflammation and oxidative stress contribute to the subsequent decline of kidney function [41, 42].

The fact that the use of specific conditioning regimens did not influence the development of CKD requires attention. In our group, the frequency of use of the medications or TBI, used in the pretransplantation phase, did not differ significantly according to CKD status.

This may be due to the improvement of the posttransplantation hematologic and renal care that took place over the past three decades, and currently used conditioning regimens are associated with less toxicity [43].

The fact that 24.5% of the HSCT patients developed CKD, but that this did not materially adversely influence survival, is an important observation. Previously, the development of CKD was found to be an important risk factor for mortality in children after HSCT [6, 22, 44]. In our group, the development of CKD was frequent; however, the deterioration of eGFR was moderate (Figure 1). This, along with the fact that the patients have been followed in a tertiary care setting by a center that includes both high-standard hematology and renal departments, may have been protective against excess mortality in the CKD group (Figure 2).

Several studies investigated the factors associated with CKD and its progression in the pediatric population at large. These included most frequently hypertension and proteinuria. Of other possible factors, the progression of the background disease, the use of nephrotoxic medications, sex, age, ethnicity, other genetic factors, urological problems, especially the congenital anomalies of kidney and urinary tract (CAKUT), and comparatively worse social status have been mapped [45, 46, 47, 48]. In our study, we analyzed factors associated with CKD in a group of post-HSCT pediatric patients, in whom, due to the peculiarities of the indications for transplantation, the peritransplant procedures, and the comorbidities, the factors associated with CKD may have differed from those in other studies.

Several limitations of our study require a cautious approach to the results we present. First, our study was a retrospective analysis of available data from all patients, and no a priori sample size calculation was performed. However, in comparison to other similar studies, our group is of similar size, and most of the relations we report are robust even after considering their post hoc nature. Further, the term “pediatric population” refers to a heterogenous group of patients of very different body sizes and compositions and the ongoing physiological changes. Moreover, renal function in infants and small children remains difficult to assess as it reaches the steady state around the age of 2 years. Therefore, eGFR in some cases may be overestimated. The 24-h urine collections were rarely obtained as they require patients' cooperation and, in small children, bladder catheterization, which was avoided for this indication. Also, we included patients who underwent HSCT over the span of 15 years. During such time, treatment approaches to diseases constituting indications for HSCT, the peritransplantation procedures, and posttransplantation care, including renal follow-up, may have changed significantly. However, we tried to limit that drawback by selecting patients who were transplanted after 2004, whose care we did not expect to differ significantly. In other studies, the time required to accumulate numbers of patients like our group required the enrolment of patients who underwent HSCT over a similar timespan.

In our analyses, we based the diagnosis of CKD on eGFR, proteinuria, urinary abnormalities, and abnormalities detected in imaging studies. However, the numbers of persons with CKD we present are in line with those reported by other researchers.

Finally, our study is a single-center retrospective analysis, and no causality can be inferred. However, the 5-year follow-up period is comparatively long, and our hospital is a high-volume pediatric center serving the population of South-East Poland of approximately 8 million inhabitants.

In conclusion, CKD in the aftermath of HSCT is a common complication. The current standard hematologic periprocedural care does not seem to adversely influence its incidence. Neither infection nor other organ dysfunction was significantly related to CKD status. Even though the baseline eGFR in CKD patients was lower than in non-CKD patients, its level was not related to the development of CKD after other confounders were accounted for. This differs from the observations of other authors and the view that a lower baseline eGFR at the time of transplantation is a risk factor for CKD.

The development of AKI, higher AKI KDIGO grade, hypertension, malnutrition, iron overload, and diabetes mellitus was strongly associated with post-HSCT follow-up CKD. Female sex was protective. This underlines the need for particular attention to patients' nutrition, prevention and treatment of iron overload, and adequate blood pressure and diabetes control in pediatric patients after HSCT.

A multidisciplinary medical team (including a transplantologist, hemato-oncologist, and nephrologist) experienced in the care of the pediatric population is the key to high-standard medical care for post-HSCT patients. This would facilitate the prevention of further kidney damage in the first years following transplantation despite facing various risk factors and complications. Further studies are needed to translate our results into daily clinical practice.

## Figures and Tables

**Table 1:** The characteristics of the study group.

**Characteristic**	**CKD (** **n** = 49**)**	**No CKD (** **n** = 151**)**	**p**
Age, median (p5–p95) (years)	10.3 (0.7–18.0)	8.3 (0.6–17.0)	0.25
Sex, males (%)	75.5 (37)	63.6 (96)	0.12
Weight, median (p5–p95) (kg)	29.7 (6.4–75.0)	26.2 (6.5–63.9)	0.55
Creatinine, median (p5–p95) (*μ*mol/L)	36.5 (14.1–95.0)	33.3 (17.5–56.7)	0.17
Urea, mean (SD) (mmol/l)	4.1 (2.1)	3.4 (1.4)	0.04
WBC, median (p5–p95) (thousand/*μ*L)	5.6 (1.0–7.8)	3.58 (0.53–9.5)	0.05
Cys C, mean (SD) (mg/L)	0.88 (0.40)	0.75 (0.41)	0.1
Hb, mean (SD) (g/dL)	10.4 (2.0)	10.5 (1.6)	0.95
PLT, median (p5–p95) (thousand/*μ*L)	118.0 (28.0–480.0)	182.5 (9.0–448.0)	0.29
Glucose, mean (SD) (mmol/L)	5.2 (1.5)	4.9 (0.9)	0.22
AST, median (p5–p95) (IU/L)	25.3 (12.7–92.6)	39.1 (14.2–102.6)	0.01
ALT, median (p5–p95) (IU/L)	34.0 (17.7–124.4)	39.2 (15.4–137.0)	0.39
Total protein, mean (SD) (g/L)	60.9 (7.8)	60.9 (9.7)	0.98
Albumin mean (SD) (g/L)	35.8 (6.3)	36.3 (6.9)	0.62
Follow-up, mean (SD) (years)	4.2 (1.7)	4.1 (1.8)	0.71
eGFR, baseline, mean (SD) (mL/min/1.73 m^2^)	119.7 (35.8)	137.2 (35.7)	0.003
eGFR at transplant mean (SD)	120.2 (47.7)	150.6 (43.7)	< 0.0001
6 months, mean (SD)	98.9 (37.8)	134.5 (39.5)	< 0.0001
1 year, mean (SD)	101.7 (38.6)	138.4 (39.8)	< 0.0001
2 years, mean (SD)	108.9 (35.8)	129.5 (25.9)	0.001
3 years, mean (SD)	115.0 (37.4)	126.8 (29.6)	0.04
4 years, mean (SD)	112.2 (33.9)	121.2 (24.3)	0.13
5 years, mean (SD)	108.2 (33.1)	119.6 (24.8)	0.05

Abbreviations: AKI, acute kidney injury; ALT, alanine transferase; AST, aspartate transferase; CKD, chronic kidney disease; CysC, cystatin C; eGFR, estimated glomerular filtration rate; Hb, hemoglobin; PLT, platelets; WBC, white blood count.

**Table 2:** The characteristics associated with HSCT.

**Characteristic**	**CKD (** **n** = 49**)**	**No CKD (** **n** = 151**)**	**p**
ATG (%) (*n*)	8.2 (4)	8.6 (13)	0.92
Busulphan (%) (*n*)	30.6 (15)	41.1 (62)	0.19
Alemtuzumab (%) (*n*)	2.0 (1)	2.0 (3)	0.98
Cyclophoshamide (%) (*n*)	38.8 (19)	42.4 (64)	0.66
Fludarabine (%) (*n*)	40.8 (20)	34.4 (52)	0.42
Melphalan (%) (*n*)	18.4 (9)	9.9 (15)	0.11
Total body irradiation (%) (*n*)	32.7 (16)	31.8 (48)	0.91
Thiotepa (%) (*n*)	8.2 (4)	9.3 (14)	0.81
Treosulfan (%) (*n*)	10.2 (5)	11.3 (17)	0.84
Etoposide (%) (*n*)	36.7 (18)	32.5 (49)	0.58
Source of cells			0.42
Bone marrow (%) (*n*)	14.3 (7)	11.3 (17)	
Peripheral blood stem cells (%) (*n*)	81.6 (40)	86.8 (131)	
BM + PBSC (%) (*n*)	0 (0)	0.7 (1)	
Stem cells (%) (*n*)	2.0 (1)	0 (0)	
Cord blood stem cells (%) (*n*)	2.0 (1)	1.3 (2)	
Donor type			0.28
Family (%) (*n*)	40.8 (20)	32.5 (49)	
Unrelated (%) (*n*)	59.2 (29)	67.5 (102)	
CsA (%) (*n*)	93.9 (46)	95.4 (144)	0.68
Tacrolimus (%) (*n*)	4.1 (2)	2.0 (3)	0.41
Primary disease			
Leukemia (%) (*n*)	44.9 (22)	49.0 (74)	0.62
Lymphoma (%) (*n*)	6.1 (3)	4.0 (6)	0.53
Solid tumor (%) (*n*)	2.0 (1)	0.7 (1)	0.40
Bone marrow insufficiency (%) (*n*)	18.4 (9)	20.5 (31)	0.74
Immunodeficiency (%) (*n*)	18.4 (9)	20.5 (31)	0.74
Other disease (%) (*n*)	8.2 (4)	4.0 (6)	0.24

Abbreviations: ATG, antithymocyte globulin; BM, bone marrow; CKD, chronic kidney disease; CsA, cyclosporin A; PBSC, peripheral blood stem cells.

**Table 3:** Complications post-HSCT.

**Characteristic (%) (** **n** **)**	**CKD**	**No CKD**	**p**
AKI			< 0.0001
None	38.8 (19)	82.1 (124)	< 0.0001
KDIGO 1	18.4 (9)	7.3 (11)	0.02
KDIGO 2	20.4 (10)	6.0 (9)	0.003
KDIGO 3	22.5 (11)	4.6 (7)	0.0002
Hemorrhagic cystitis	12.2. (6)	14.6 (22)	0.68
GVHD	69.4 (34)	81.5 (123)	0.07
Glucksberg			0.21
0	34.7 (17)	18.5 (28)	
1	34.7 (17)	43.1 (65)	
2	16.3 (8)	23.2 (35)	
3	10.2 (5)	9.9 (15)	
4	4.1 (2)	5.3 (8)	
cGVHD			0.85
0	49.0 (24)	55.0 (83)	
1	26.5 (13)	21.9 (33)	
2	16.3 (8)	17.2 (26)	
3	6.1 (3)	5.3 (8)	
4	2.0 (1)	0.7 (1)	
CMV	42.9 (21)	24.5 (37)	0.01
EBV	18.4 (9)	14.6 (22)	0.52
Other viruses	42.9 (21)	33.1 (50)	0.22
Fungal infections	18.4 (9)	10.7 (16)	0.16
*Clostridium difficile*	6.1 (3)	8.6 (13)	0.58
Heart failure	6.1 (3)	2.0 (3)	0.14
VOD	4.1 (2)	6.0 (9)	0.62
Iron overload	34.7 (17)	15.9 (24)	0.005
Osteoporosis	6.1 (3)	2.0 (3)	0.14
Adrenal insuff.	26.5 (13)	11.3 (17)	0.009
Hypothyroidism	26.5 (13)	8.6 (13)	0.001
Liver dysfunction	53.1 (26)	45.7 (69)	0.37
DM	18.4 (9)	8.0 (12)	0.04
Pancreatitis	16.3 (8)	10.6 (16)	0.28
Malnutrition	44.9 (22)	21.2 (32)	0.001
Sepsis	34.7 (17)	25.2 (38)	0.19
ICU	28.6 (14)	21.9 (33)	0.34

Abbreviations: AKI, acute kidney injury; cGVHD, chronic graft-versus-host disease; CKD, chronic kidney disease; CMV, cytomegalovirus; DM, diabetes mellitus; EBV, Epstein–Barr virus; GVHD, graft-versus-host disease; ICU, intensive care unit admission; VOD, venoocclusive disease.

**Table 4:** Cox regression analysis of time to development of CKD. Age, sex, and eGFR were forced into the model. The remaining variables were selected using a stepwise procedure.

**Parameter**	**p** **value**	**RHR**	**95% CI**	
Age (years)	0.77	1.01	0.95	1.08
Female sex	0.0008	0.20	0.08	0.51
eGFR_base (mL/min/1.73 m^2^)	0.51	1.00	0.99	1.01
CMV (present vs. absent)	0.05	2.24	1.01	4.95
AKI_KDIGO (class)	< 0.0001	2.36	1.76	3.16
Adrenal insufficiency (present vs. absent)	0.027	0.26	0.08	0.86
DM (present vs. absent)	0.0003	4.12	1.91	8.92
Malnutrition (present vs. absent)	0.002	3.57	1.61	7.92
Iron overload (present vs. absent)	0.78	1.01	0.95	1.08

Abbreviations: AKI_KDIGO, acute kidney injury stages (0–3); AKI_KDIGO (class), AKI KDIGO classification; CKD, chronic kidney disease; CMV, cytomegalovirus infection; DM, diabetes mellitus; eGFR, estimated glomerular filtration rate; eGFR_base, eGFR baseline; RHR, relative hazard ratio.

## Data Availability

The data can be made available upon reasonable request.
